# High Diversity and Functional Potential of Undescribed “Acidobacteriota” in Danish Wastewater Treatment Plants

**DOI:** 10.3389/fmicb.2021.643950

**Published:** 2021-04-22

**Authors:** Jannie Munk Kristensen, Caitlin Singleton, Lee-Ann Clegg, Francesca Petriglieri, Per Halkjaer Nielsen

**Affiliations:** Department of Chemistry and Bioscience, Center for Microbial Communities, Aalborg University, Aalborg, Denmark

**Keywords:** Acidobacteriota, metagenomics, FISH-probe design, MAGS, wastewater treatment plants

## Abstract

Microbial communities in water resource recovery facilities encompass a large diversity of poorly characterized lineages that could have undescribed process-critical functions. Recently, it was shown that taxa belonging to “Acidobacteriota” are abundant in Danish full-scale activated sludge wastewater treatment plants (WWTP), and here we investigated their diversity, distribution, and functional potential. “Acidobacteriota” taxa were identified using a comprehensive full-length 16S rRNA gene reference dataset and amplicon sequencing surveys across 37 WWTPs. Members of this phylum were diverse, belonging to 14 families, eight of which are completely uncharacterized and lack type strains. Several lineages were abundant, with relative abundances of up to 5% of the microbial community. Genome annotation and metabolic reconstruction of 50 high-quality “Acidobacteriota” metagenome-assembled genomes (MAGs) from 19 WWTPs showed high metabolic diversity and potential involvement in nitrogen and phosphorus removal and iron reduction. Fluorescence *in situ* hybridization (FISH) using newly-designed probes revealed cells with diverse morphologies, predominantly located inside activated sludge flocs. FISH in combination with Raman microspectroscopy revealed ecophysiological traits in probe-defined cells from the families *Holophagaceae*, *Thermoanaerobaculaceae*, and *Vicinamibacteraceae*, and families with the placeholder name of midas_f_502, midas_f_973, and midas_f_1548. Members of these lineages had the potential to be polyphosphate-accumulating organisms (PAOs) as intracellular storage was observed for the key compounds polyphosphate and glycogen.

## Introduction

Wastewater treatment plants (WWTP) with biological nutrient removal have the primary role of removing nutrients and pollutants from wastewater to protect the environment. These systems are increasingly acknowledged as resource recovery facilities with an important role in resource recovery such as clean water, bioenergy, and phosphorus (Nielsen, 2017). Complex microbial communities are responsible for key biological functions, but many bacterial lineages are still undescribed at the taxonomic, genomic, and functional level (Nierychlo et al., 2020). Most characterized taxa in activated sludge WWTPs across the world are from the phyla Proteobacteria, Actinobacteriota, Bacteroidota, Spirochaetota, and Nitrospirota (Wu et al., 2019), but other less well-described phyla are also observed as abundant. For example, almost nothing is known about the potential roles of abundant “Acidobacteriota” members in the wastewater treatment process and only a few studies have visualized (McIlroy et al., 2015a) or evaluated the abundance of “Acidobacteriota” *in situ* (Juretschko et al., 2002; McIlroy et al., 2015b; Saunders et al., 2016; Kim et al., 2019).

The “Acidobacteriota” is a diverse phylum currently comprising 26 subdivisions (Dedysh and Yilmaz, 2018), with the most information available for subdivision 1 (Kielak et al., 2016). Most “Acidobacteriota” have been investigated in soil and sediment, such as agricultural soils (Navarrete et al., 2013), forest soils (Štursová et al., 2012), arctic tundra (Ivanova et al., 2020), deserts (Gupta et al., 2015; Armstrong et al., 2016), and peat soils (Hausmann et al., 2018), while only a few are investigated in water systems (Farag et al., 2014). Culture-independent studies have recently expanded the number of metagenome-assembled genomes (MAGs), enhancing the available knowledge for the entire phylum in different soil environments (Hausmann et al., 2018; Woodcroft et al., 2018), and providing some insight into the functional potential of a few subdivisions.

The “Acidobacteriota” encompasses both anaerobes and aerobes, and members from certain subdivisions have special physiological traits such as iron reduction and fermentative growth (Coates et al., 1999), and phototrophy (Bryant et al., 2007). Some also have the ability to grow under thermophilic conditions (Eichorst et al., 2018). Two species have been isolated from water samples, *Geothrix fermentans* DSM14018 from subdivision 8 and *Thermoanaerobaculum aquaticum* MP-01 from subdivision 23. Both lineages are strict anaerobes (Coates et al., 1999; Losey et al., 2013), which is in contrast to the majority of known “Acidobacteriota” described as aerobic or microaerophilic (Eichorst et al., 2018). *G. fermentans* is involved in the degradation of aromatic hydrocarbons, can grow fermentatively on citrate or fumarate, and can oxidize long-chain fatty acids such as palmitate with Fe(III) (Coates et al., 1999). *T. aquaticum* grows at a temperature optimum of 60°C and is also capable of fermentative growth as well as reducing Fe(III) and Mn(IV) (Coates et al., 1999; Losey et al., 2013).

Diversity studies have been hampered by the general lack of representative full-length 16S rRNA gene sequences in the reference databases, but a new approach to generate millions of high-quality full-length reference sequences has changed this (Karst et al., 2018). Using this method, an ecosystem-specific reference database for wastewater treatment systems containing more than 9,500 full-length high-quality 16S rRNA gene sequences has been established, describing the majority of diversity in Danish WWTPs (Dueholm et al., 2020) including the “Acidobacteriota”. By applying the new taxonomy provided by MiDAS 3 (Nierychlo et al., 2020), including placeholder names for novel taxa generated using AutoTax (Dueholm et al., 2020) with identity thresholds of 98.7, 94.5, and 86.5% for species, genus, and family, respectively (Yarza et al., 2014). The “Acidobacteriota” can with this approach be studied at high resolution (species-level) in amplicon studies (Dueholm et al., 2020), and the near-complete reference database also allows probe design for fluorescence *in situ* hybridization (FISH) and additional ecophysiological investigations.

Here, we present a comprehensive study of the abundance and distribution of “Acidobacteriota” in 37 Danish full-scale activated sludge plants with nutrient removal. The diversity was described using the comprehensive set of full-length 16S rRNA exact amplicon sequences (FL-ASVs) and MiDAS taxonomy, and their distribution determined by 16S rRNA amplicon sequencing. The full-length 16S rRNA gene sequences were also used to design a novel set of FISH probes to visualize morphology and spatial arrangements of cells for abundant taxa. Raman microspectroscopy was applied to characterize key physiological traits, such as presence of storage polymers in probe-defined members of abundant genera. In addition, we linked the full-length 16S rRNA gene sequences to 50 high-quality MAGs, recently retrieved from the same WWTPs (Singleton et al., 2021), to provide insight into “Acidobacteriota” functional potential.

## Materials and Methods

### Abundance Determination of “Acidobacteriota” by Amplicon Sequencing

Survey of the microbial communities was carried out using activated sludge from 37 full-scale Danish WWTP with nutrient removal (nitrogen and phosphorus), sampled 2–4 times a year over 7 years (2011–2017). Sampling, DNA extraction, library preparation, and sequencing were performed as described by Stokholm-Bjerregaard et al. (2017). For extraction of DNA, the sample was used for direct extraction of DNA with the FastDNA^®^ Spin Kit for Soil (MP Biomedicals, CA, United States), with the modification of bead beating to be extended to 4 × 40 s at 6 m/s instead of 40 s at 6 m/s (Albertsen et al., 2015). Nucleic acids were quantified using dsDNA BR Assay Kit on a Qubit 2.0 Fluorometer (Invitrogen, CA, United States). The 16S V1–3 library preparation was performed as described by Albertsen et al. (2015) and McIlroy et al. (2015b), modified from Caporaso et al. (2011) with the use of bacterial primers, which amplify a DNA fragment of 500 bp of the V1–3 region of the 16S rRNA gene with primers [27F (AGAGTTTGATCCTGGCTCAG) and 534R (ATTACCGCGGCTGCTGG)]. The library DNA concentration was measured with dsDNA HS Assay Kit on a Qubit 2.0 Fluorometer (Invitrogen, CA, United States), and the quality was validated using TapeStation 2200 using D1K screentapes (Agilent Technologies, CA, United States). Libraries were pooled in equimolar concentrations. The library pool was paired-end (2 × 300 bp) sequenced on a MiSeq (Illumina, CA, United States) using MiSeq Reagent kit v3 (Illumina, CA, United States). All sequenced libraries were screened for PhiX contamination. The reads were dereplicated and formatted for use in the USEARCH UNOISE workflow (Edgar, 2016b). The dereplicated reads were used to generate amplicon sequencing variants, ASVs, using the USEARCH v. 10 unoise3 with default settings. Taxonomy was assigned using SINTAX classifier (Edgar, 2016a) as implemented in USEARCH v. 10, using the SILVA taxonomy v. 138 (Quast et al., 2013), and for *de novo* taxa, MiDAS 3 (Nierychlo et al., 2020) identifiers (placeholder names) have been added. The results were analyzed in R Core Team (2018) using the Rstudio IDE^1^ and the ampvis2 R package v. 2.4.2.1^2^.

### 16S rRNA Gene Phylogenetic Analysis and FISH Probe Design

Phylogenetic analysis and FISH probe design were performed using the software ARB v. 6.0.6 (Ludwig et al., 2004). By analysis of the FL-ASVs found in activated sludge (Dueholm et al., 2020), a phylogenetic tree was calculated with IQ-TREE v. 1.5.6 (Nguyen et al., 2015) using RAxML GTR algorithm with 1,000 bootstraps and displayed with iTOL v. 4.4.2 (Letunic and Bork, 2019). For abundant groups, 10 FISH probes were designed. The probes were validated *in silico* with mathFISH (Yilmaz et al., 2011) to test the *in silico* hybridization efficiency of target and non-target sequences. The number of non-target sequences with 0, 1, and 2 mismatches were assessed using the probe match function in ARB and the mismatch analysis function in mathFISH. Unlabeled competitor probes were designed for mismatching non-target organisms which were not possible to discriminate with a higher formamide % (Yilmaz et al., 2008). All probes were purchased from biomers (Biomers.net, Ulm, Germany) and were labeled with 5(6)-carboxyfluorescein-*N*-hydroxysuccinimide ester (FLUOS) or indocarbocyanine (Cy3) fluorochromes. The optimal hybridization formamide concentration was found using formamide dissociation curves, of increasing % of formamide from 0 to 70% in 5% increments, thereafter relative fluorescent signal of 50 cells were measured with the software ImageJ (v 1.52a). From the calculated average values, a curve was made and values right before a decline on the curve determined as the optimal formamide concentration. Where available, pure cultures obtained as growing cultures from DSMZ (Braunschweig, Germany) were fixed upon arrival and used to optimize the curves, otherwise an environmental sample of activated sludge with a high amplicon abundance was used. When possible, hierarchical probes were applied (*Holophagaceae* and *Geothrix*, *Vicinamibacteraceae*, and genus Mb2424). *Blastocatella fastidiosa* DSM 25172 was used to optimize the probe Blasto_312, *Rhodospirillum rubrum* DSM 467 was used for competitor test on Geo_662_c2, and *Clostridium pasteurianum* DSM 525 was used to test the need of competitor for Holo_1154_c1.

Growing pure cultures obtained from DSMZ (Braunschweig, Germany) and activated sludge samples were fixed with 4% paraformaldehyde (final concentration) for 3 h at 4°C and washed three times with 1 ml of sterile filtered tap water. FISH was performed as described by Wagner et al. (2003) with 3 h hybridization to obtain sufficient fluorescent signal. A nonsense NON-EUB probe was applied to samples as negative control for binding. Quantitative FISH was performed using the software DAIME (Daims et al., 2006). Abundances of the general community were measured as the percentage of area with EUB-mix (Daims et al., 1999). A white light laser confocal microscope (Leica TCS SP8 X, Wetzlar), with 63× or 100× magnification objectives, was used to capture FISH images.

### Raman Microspectroscopy

Probe-defined cells in activated sludge samples and the two pure cultures *B. fastidiosa* DSM 25172 and *G. fermentans* DSM14018 (DSMZ, Braunschweig, Germany) were investigated for the presence of storage polymers [poly-P, polyhydroxyalkanoates (PHA), and glycogen] with Raman microspectroscopy. Spectra from single cells were obtained using a Horiba LabRam HR 800 Evolution (Jobin Yvon–France) equipped with a Torus MPC 3000 (United Kingdom) 532 nm 341 mW solid-state semiconductor laser. The Raman spectrometer was calibrated prior to obtaining all measurements to the first-order Raman signal of Silicon, occurring at 520.7 cm^–1^. The CaF_2_ Raman substratum also contains a single-sharp Raman marker at 321 cm^–1^, which serves as an internal reference point in every spectrum. The incident laser power density on the sample was attenuated down to 2.1 mW/μm^2^ using a set of neutral density (ND) filters (5%). The Raman system is equipped with an in-built Olympus (model BX-41) fluorescence microscope. A 50×, 0.75 numerical aperture dry objective (Olympus M Plan Achromat, Japan) with a working distance of 0.38 mm was used throughout the work. A diffraction grating of 600 mm/groove was used and the Raman spectra collected spanned the wavenumber region of 200–1,800 cm^–1^. The slit width of the Raman spectrometer and the confocal pinhole diameter were set, respectively, to 100 and 72 μm. Raman spectrometer operation and subsequent processing of spectra were conducted using LabSpec version 6.4 software (Horiba Scientific, France). All spectra were baseline corrected using a sixth order polynomial fit.

### Retrieval of Genomes and Phylogenetic Classification

“Acidobacteriota” MAGs from Singleton et al. (2021) were classified taxonomically using GTDB-Tk (Chaumeil et al., 2019) v1.3.0, with species representatives determined using 95% average nucleotide clustering and dRep (Olm et al., 2017) v2.3.2. A phylogenetic genome tree of the “Acidobacteriota” MAGs, with five Chloroflexota genomes used as an outgroup, was produced using the concatenated alignment of 120 single copy proteins from GTDB-Tk. The protein alignment was used as input for IQ-TREE v2.0 (Nguyen et al., 2015) and the maximum likelihood tree was created using the WAG + G model with 100 bootstrap iterations. The MAGs were annotated using EnrichM v0.5.0^3^ to annotate against the KEGG orthology (KO) (Kanehisa et al., 2019) annotated uniref100 database. The annotated genomes were searched for key KO terms representing pathways of interest. Prokka v1.14 (Seemann, 2014) was also used to annotate the genomes and provide protein sequences to identify CXXCH motifs in multiheme cytochromes using a python script^4^. Infernal v1.1.2 (Nawrocki and Eddy, 2013) was used alongside BEDTools v2.27 (Quinlan and Hall, 2010) to retrieve the 16S rRNA genes for comparison to the MiDAS 16S rRNA gene sequences as in Singleton et al. (2021). BLAST against NCBI nr was used to confirm genes of interest (Camacho et al., 2009).

## Results and Discussion

### Distribution and Abundance of Members of “Acidobacteriota” in Danish Full-Scale WWTP With Nutrient Removal

**FIGURE 1:**
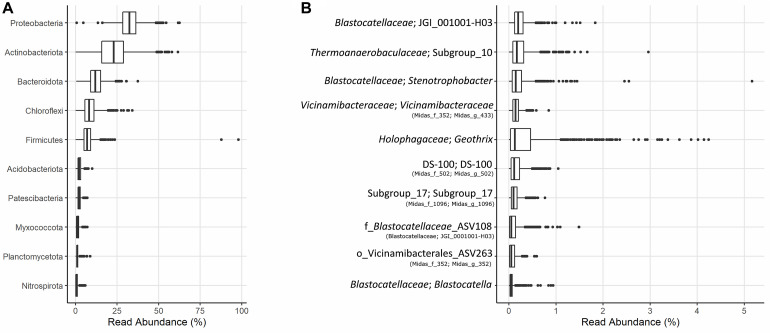
**(A)** Boxplot of the relative abundances of the 10 most abundant phyla in 37 Danish activated sludge plants (2006–2017). **(B)** Boxplot of the 10 most abundant genera of “Acidobacteriota” found in the same plants, and their respective families or nearest known taxa. The MiDAS 3 placeholder name from the MiDAS taxonomy is shown in parentheses.

**FIGURE 2:**
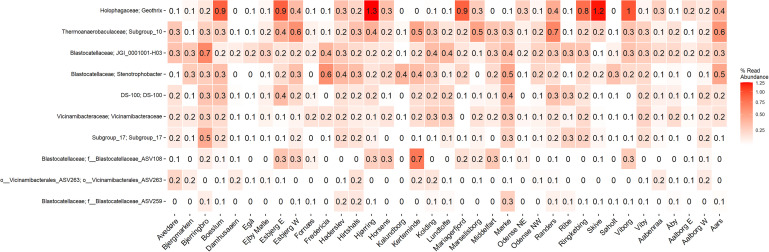
A heatmap showing the average abundance distribution of the top 10 most abundant genera of “Acidobacteriota” in 21 Danish wastewater treatment plants.

The microbial community of 37 full-scale Danish WWTPs with nutrient removal was determined using 16S rRNA gene amplicon sequencing and SILVA138 taxonomy. The overall distribution of bacteria revealed Proteobacteria and “Actinobacteriota” as the most abundant phyla, with “Acidobacteriota” the sixth most abundant on average (Figure 1A). “Acidobacteriota” populations were widely spread and abundant across all Danish WWTPs in abundances of up to 5% with average read abundances of 0.7–2.9%. The distribution of the 10 most abundant genera of “Acidobacteriota” showed a high diversity and variation by sample. On average, a genus-level clade from the *Blastocatellaceae* family with the placeholder name “JGI_0001001-H03” was most abundant followed by “Subgroup 10” from *Thermoanaerobaculaceae* (Figure 1B). These two genera were found abundant (more than 0.1% relative abundance) in all WWTPs (Figure 2). The third and fourth most abundant genera were *Stenotrophobacter* and an unknown genus from the family *Vicinamibacteraceae*. Genus *Geothrix* from the *Holophagaceae* family was the most abundant “Acidobacteriota” member in several WWTPs (Hjørring, Skive, and Viborg, Figure 2). The remaining top 10 genera were from unknown families and genera.

Few studies have examined “Acidobacteriota” in WWTPs, and only a couple of key populations have been explored in detail. *Geothrix* is recognized as an abundant lineage in nutrient removal WWTPs in South Korea and Vietnam (Kim et al., 2019), suggesting *Geothrix* to be metabolically active and growing in the plants. In a study by Saunders et al. (2016), an unknown genus from the “Acidobacteriota” order with the placeholder name “Sva0725” was defined as a member of the abundant core genera also suggesting them growing in the plants and influencing carbon turnover. However, little is known about the *in situ* activity of “Acidobacteriota” and their possible role in nitrogen or phosphorus cycling.

### High Diversity of Novel “Acidobacteriota”

The diversity of “Acidobacteriota” in the WWTPs was explored using the ecosystem-specific MiDAS 3 reference database and related MiDAS 3 taxonomy (Dueholm et al., 2020; Nierychlo et al., 2020), and MAGs. A total of 347 full-length 16S rRNA gene sequences belonging to “Acidobacteriota” were retrieved, 282 from the MiDAS 3 reference database, and 65 were from 50 high-quality MAGs obtained from Danish WWTPs (Singleton et al., 2021).

**FIGURE 3:**
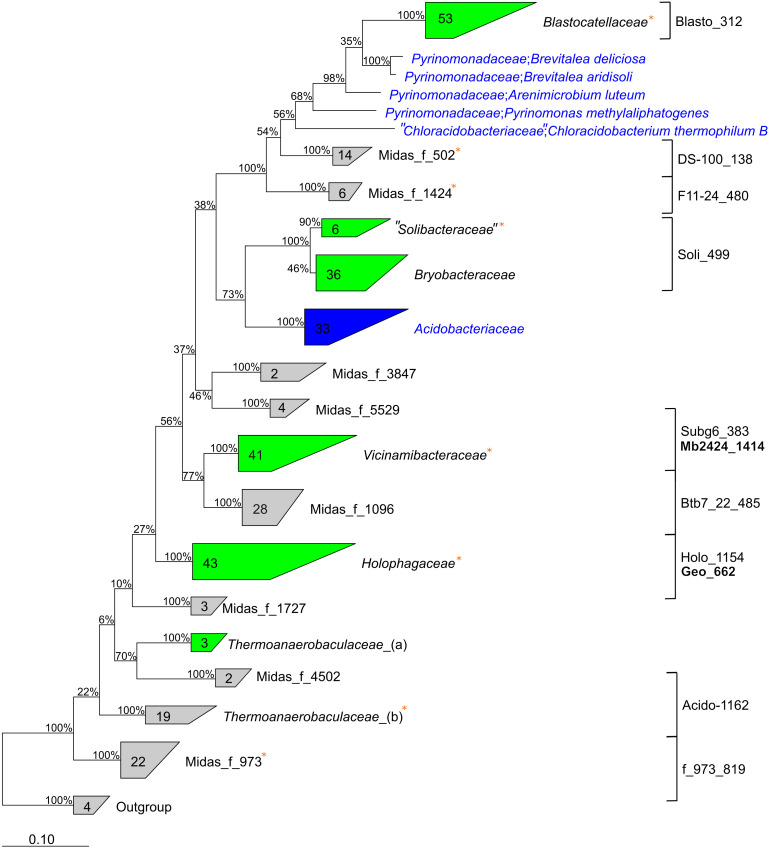
Phylogenetic 16S rRNA gene tree showing the members of the “Acidobacteriota” found in activated sludge combined with reference sequences from the SILVAv. 138 database (left). Labels show the family names of “Acidobacteriota”. Unknown *de novo* families are displayed with *de novo* MiDAS 3 placeholder names. Groups in gray color contain only MiDAS 3 reference database sequences, blue groups contain only sequences from SILVAv. 138, and green groups contain a combination of MiDAS and SILVAv. 138 sequences. MiDAS 3 sequences from the phylum Nitrospirota were used as an outgroup. Bootstrap values for 1,000 replicates are indicated by percentage values. Coverage of newly designed FISH probes is indicated to the right and clades where metagenome-assembled genomes (MAGs) were retrieved are indicated by an orange star.

Phylogenetic analyses using the MiDAS 3 taxonomy showed the “Acidobacteriota” lineages represented 114 species from 44 different genera in 14 different families (Figure 3). The families are located within eight different subdivisions of “Acidobacteriota”, and only six families are described by the SILVA138 taxonomy: *Blastocatellaceae*, *Vicinamibacteraceae*, “*Solibacteraceae*,” *Bryobacteraceae*, *Holophagaceae*, and *Thermoanaerobaculaceae*. Thus, nearly all well-characterized families (by SILVA taxonomy) of “Acidobacteriota” were found in the WWTPs, except the common soil lineage of the *Acidobacteriaceae*. The 16S rRNA gene sequences within eight novel family-level clades were given MiDAS placeholder names according to the MiDAS 3 taxonomy using AutoTax (Dueholm et al., 2020). This revealed eight novel family-level clades for which there are no representative reference sequences in SILVA, suggesting specific novel lineages were involved in wastewater treatment (Figure 3). It also shows the advantage of using AutoTax, as these family level clades (and genera and species) have robust placeholder names that can now be recognized across studies until valid names are given.

To determine how the 50 “Acidobacteriota” HQ MAGs, retrieved from a subset of the WWTPs investigated here (Singleton et al., 2021), compared to the diversity of described “Acidobacteriota” worldwide, we compared the 16S rRNA gene sequences to the SILVA138 Ref NR database (Supplementary Figure 1). The 16S rRNA gene sequences with genome classification from Genome Taxonomy Database (GTDB) (Parks et al., 2020) mostly had the same taxonomic classification as the SILVA138 Ref NR database. The novel lineages clustered similarly to the 16S rRNA gene sequences obtained using AutoTax, which further supports the robustness of the method. More detailed phylogenetic analyses were carried out to investigate the 16S rRNA gene identity for the defined families and for comparison with the genome taxonomy of the recovered MAGs classified through GTDB (Supplementary Table 1). The 50 high-quality “Acidobacteriota” MAGs were found to belong to 34 different species [<95% whole genome average nucleotide identity (ANI)] and the classes “*Acidobacteriae*” (3 MAGs), *Blastocatellia* (31), *Holophagae* (8), *Thermoanaerobaculia* (7), and the *Vicinamibacteria* (1). Four of the MAGs were recovered as circular closed genomes, 3 are separate species within *Blastocatellia*, and 1 belongs to *Thermoanaerobaculia*. The MAGs represented 8 of the 14 “Acidobacteriota” families found in MiDAS 3.

The *Holophagaceae* and *Thermoanaerobaculaceae* had identical classifications for both genome-based and 16S rRNA gene-based taxonomic approaches. However, MAGs with 16S rRNA genes placing them within the *Blastocatellaceae* and “*Solibacteraceae*” were classified as *Pyrinomonadaceae* and *Bryobacteraceae* at the genome level, highlighting taxonomic discrepancies between 16S rRNA gene-based and genome-based classification methods (Parks et al., 2020).

The WWTP “Acidobacteriota” MAGs were compared to the GTDB RefSeq release 95 genomes used during the genome-based classification process (Supplementary Table 2). This revealed that 49 of the MAGs belong to novel species that had no other MAGs or isolate genomes in the database. Furthermore, 45 and 17 MAGs belonged to novel genera and families, respectively, with no other formally named MAG or isolate present in the database. Three placeholder families had novel taxonomic classifications for both 16S rRNA gene and genome-based classification methods (midas_f_502, midas_f_973, and midas_f_1424). This highlights the extent of novel diversity within the phylum, and the void of knowledge regarding “Acidobacteriota” in activated sludge systems.

### *In situ* Visualization of the 10 Most Abundant “Acidobacteriota” in Danish Activated Sludge Plants

Fluorescence *in situ* hybridization probes were designed for visualization and FISH-Raman studies of the 10 most abundant “Acidobacteriota” families (Table 1), as evaluated by amplicon sequencing. Due to the high similarity of the 16S rRNA genes between some genera, especially in the *Blastocatellaceae* family (Figure 3), the FISH probes were mainly designed to target at the placeholder family level or family level (7), although three genus-specific probes were possible: *Geothrix* in *Holophagaceae*, midas_g_1096 in Subgroup 17 (midas_f_1096), and midas_g_433 in *Vicinamibacteraceae*. In addition, the probe targeting the *Thermoanaerobaculaceae* Subgroup 10 described by McIlroy et al. (2015a) was used. The probes were designed based on the *de novo* taxonomy assigned by AutoTax.

**TABLE 1:** Details of the designed Fluorescence *in situ* hybridization (FISH) probes.

Probe	*Escherichia coli* pos.	Target family	Target genus	Coverage*	Coverage* with 1 mismatch	Coverage* with 2 mismatches	Sequence (5′–3′)	(FA)%	Validated against	Study
Blasto_312	312–334	*Blastocatellaceae*	–	43/46 (0)	46/46 (0)	46/46 (0)	CCC GTT ATT CAG TGT CAG TGT G	30	*Blastocatella fastidiosa*	This study
Holo_1154	1154–1174	*Holophagaceae*	–	37/37 (0)	37/37 (226)	37/37 (238)	CGG TTA ACC CGG GCA GTC CC	35	Activated sludge	This study
Holo_1154_C1	1154–1174	Competitor for Holo_1154	–	0/0 (226)	N/A	N/A	CGG TTA ACC CGG GCA GTC TC	N/A		
Geo_662	662–685	*Holophagaceae*	*Geothrix*	18/18 (0)	18/18 (2)	18/18 (109)	ACG AGG AAT TCC ACC ACC CTC TC	30	Activated sludge	This study
Geo_662_C1	662–685	Competitor for Geo_662	–	0/0 (2)	N/A	N/A	ACC AGG AAT TCC ACC ACC CTC TC	N/A		
Geo_662_C2	662–685	Competitor for Geo_662	–	0/0 (20)	N/A	N/A	ACT GGG AAT TCC ACC ACC CTC TC	N/A		
Acido-1162	1162–1179	*Thermoanaerobaculaceae*	Subgroup 10	18/19 (0)	19/19 (0)	19/19 (7)	TCC TCC CCG ATT TCC GGG	25	–	McIlroy et al., 2015a
Acido-1162c1	1162–1179	Competitor for Acido-1162	–	0/0 (7)	N/A	N/A	TCC TCC CCG TTT TCC GGG	N/A		
Acido-1162h1	1162–1179	Helper for Acido-1162	–	N/A	N/A	N/A	GGA CTT GAC GTC ATC CCC RCC Y	N/A		
Mb2424_1414	1414–1436	Midas_f_352	Midas_g_433	09/09 (0)	09/09 (60)	09/09 (83)	CTT CTA GTA CAG CCA GCT TTC G	35	Activated sludge	This study
Mb2424_1414_C1	1414–1436	Competitor for mb2424_1414		0/0 (21)	N/A	N/A	TTT CTA GTA CAG CCA GCT TTC G	N/A		
Subg6_383	383–401	*Vicinamibacteraceae*	–	39/39 (0)	39/39 (23)	39/39 (526)	GCG TTG CGT CGT CAG GCT	25	Activated sludge	This study
F11-24_480	480–502	Midas_f_1424	–	05/06 (0)	06/06 (0)	06/06 (1)	GGG CTT ACA TAT GGT ACC GTC A	25	Activated sludge	This study
DS-100_138	138–160	Midas_f_502	–	14/14 (0)	14/14 (0)	14/14 (1)	GGA TCG TTA TTC CCC ACC CAA	40	Activated sludge	This study
DS-100_138_C1	138–160	Competitor for DS-100_138	–	0/0 (1)	N/A	N/A	GGA ACG TTA TTC CCC ACC GAA	N/A	–	
DS-100_H117	117–141	Helper probe for DS-100	–	N/A	N/A	N/A	AGG CAG ATT ACC CAC GTG TTA CTC	N/A		
DS-100_H159	159–185	Helper probe for DS-100	–	N/A	N/A	N/A	CGT TAT GCG GTA TTA GCG ACC CTT TC	N/A		
Btb7_22_485	485–503	Midas_f_1096	midas_g_1096	28/28 (0)	28/28 (20)	28/28 (346)	GGC TTC CTC CAC CGG TAC	40	Activated sludge	This study
f_973_819	819–837	Midas_f_973	–	22/22 (0)	22/22 (2)	22/22 (172)	CCG ACA CCA AGC ACC CAT	35	Activated sludge	This study
Soli_499	499–517	“*Solibacteraceae*”	–	40/40 (0)	40/40 (168)	40/40 (3971)	GCA CGT AGT TAG CCG CAG CT	35	Activated sludge	This study

*Taxonomy and coverage of groups are defined as in the MiDAS 3 reference database (Nierychlo et al., 2020). The number of non-target hits are shown in parentheses, the coverage of mismatches has been evaluated with the ARB “probe match” tool (Quast et al., 2013) and mathFISH for one or two mismatches (Yilmaz et al., 2011). Competitor probes for non-target groups were designed and used for non-target groups that could not be discriminated by higher stringency based on mathFISH mismatch analysis (Yilmaz et al., 2008).

**FIGURE 4:**
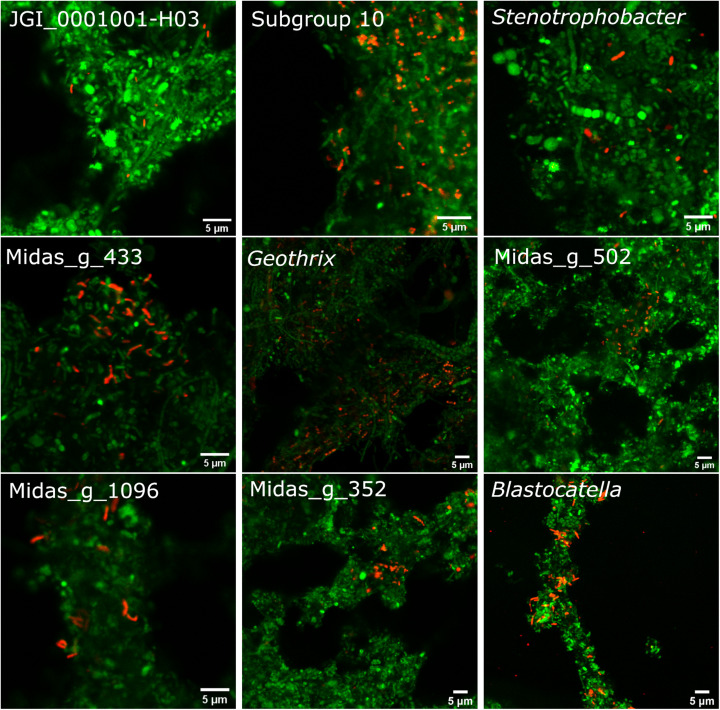
FISH images of the nine most abundant “Acidobacteriota” genera, ranked according to abundances detected by amplicon sequencing as shown on Figure 1B. Genus or family-specific probes are shown in red and the universal EUB-mix probe is shown in green. The images for the three genera, JGI_0001001-H03, *Stenotrophobacter*, and *Blastocatella* were taken using the family-specific Blasto_312 probe on samples where only that specific genus was found by amplicon sequencing. Midas_g_502 was visualized with the probe DS-100_138 targeting all sequences from midas_f_502 in the order DS-100. Midas_g_352 was imaged with the probe Subg6_383 targeting *Vicinamibacteraceae* (midas_f_502).

The 10 most abundant “Acidobacteriota” families/genera were visualized using FISH (Figure 4). Activated sludge samples with high abundances of target organisms were used for visualization and optimization of all probes except Blasto_312, where the pure culture *B. fastidiosa* DSM 25172 was used. To visualize specific genera or ASVs with family-level probes, we used samples where only the target organism(s) was present as evaluated by amplicon sequencing. The morphology of the cells was different for most probe-defined groups; some were small cocci with a diameter of 0.5–0.7 μm, in pairs or shorter chains [*Blastocatella*, DS-100 (midas_g_502), *Vicinamibacteraceae* (midas_g_433), and *Thermoanaerobaculaceae*; Subgroup 10]. The *Vicinamibacterales*_ASV_263 (midas_g_352) was found as short cocci chains or in clumps. Bacteria in the placeholder genera JGI_0001001-H03, *Stenotrophobacter*, and Subgroup 17 were small, separated, elongated rods with a length of up to 1.7 μm. Bacteria in *Geothrix* were small rods with a width of 0.3–0.5 μm growing as elongated rods of up to 5 μm long or in shorter chains. *Blastocatellaceae* were widely distributed across the activated sludge flocs in compliance with literature defining *Blastocatellaceae* species as aerobes (Foesel et al., 2013). Cells from “*Solibacteraceae*” were also found distributed over the flocs. Targeted cells from other groups were found deep inside the activated sludge flocs.

Each probe showed only one morphology, except for the Blasto_312 probe targeting *Blastocatellaceae*, which targeted both small cocci with a diameter of 0.7 μm and rods up to 1.7 μm long, indicating multiple species in the samples. Polymorphism has also been seen in pure culture of *B. fastidiosa* (Foesel et al., 2013) and may be a general feature for more species in this family. For two families, the cells were growing either as thin 0.5 μm wide, 4 μm long chains, sometimes as cocci with a diameter of 0.5–0.7 μm (midas_f_1424), or as thicker (0.9 μm) and long (up to 30 μm) filaments (“*Solibacteraceae*”) (Supplementary Figure 2).

To gain independent support of the abundances of the different “Acidobacteriota” based on 16S rRNA gene amplicon sequencing, quantification of relative biovolume fractions was also performed on a selection of activated sludge samples. The activated sludge samples were hybridized to specific probes and to the EUB338 I-III probe mix. FISH quantifications of the probe-defined “Acidobacteriota” groups were consistent with the amplicon results, indicating the lineages to be present in biovolume of up to 5% (Table 2). In general, the two independent methods for quantification gave comparable results.

**TABLE 2:** Comparison of amplicon and FISH abundances.

			Abundance%	
WWTP/target organism	Sample date	Probe	Amplicon sequencing*	qFISH
***Blastocatellaceae***				
Odense NE	2012-08-19	Blasto_312	0.4	0.1 ± 0
Fredericia	2014-02-03	Blasto_312	0.5	0.1 ± 0.1
***Holophagaceae; Geothrix***				
Hjørring	2007-06-05	Geo_662	4.2	5 ± 1.6
Skive	2006-10-28	Geo_662	2.2	2.6 ± 0.6
***Vicinamibacteraceae***				
Bjergmarken	2017-11-30	Subg6_383	0.4	0.6 ± 0.2
Avedøre	2017-09-06	Subg6_383	0.6	0.8 ± 0.2
Odense NE	2006-09-06	Subg6_383	0.2	0.6 ± 0.2
**Midas_f_1424**				
Kalundborg	2017-08-19	F11-24_480	2.6	1.3 ± 0.4
Esbjerg W	2015-08-24	F11-24_480	0.4	0.5 ± 0.3
**Midas_f_502**				
Randers	2010-08-19	DS-100_138	1.1	0.4 ± 0.2
Aalborg W	2008-10-28	DS-100_138	0.9	0.2 ± 0.1
**Midas_g_1096**				
Esbjerg W	2016-11-01	Btb_7_22_485	0.5	0.1 ± 0.1
Aalborg W	2010-10-28	Btb_7_22_485	0.5	0.1 ± 0.1
**Midas_f_973**				
Mariagerfjord	2016-09-05	F973_819	0.5	1.2 ± 0.4
Egå	2014-08-18	F973_819	0.5	0.7 ± 0.3
Lundtofte	2013-08-19	F973_819	0.8	0.5 ± 0.2
**“*Solibacteraceae*”**				
Aalborg W	2014-08-18	Soli_499	0.2	1.1 ± 0.5
Ringkøbing	2012-08-19	Soli_499	0.4	0.6 ± 0.4
Ribe	2014-11-10	Soli_499	0.2	0.9 ± 0.4

*Standard deviation on amplicon data is ≈20%.

### *In situ* Physiology as Detected by FISH-Raman

Bacterial cells from the abundant FISH probe-defined genera and families were investigated for intracellular storage polymers by Raman microspectroscopy. The accumulation of storage polymers is a metabolic strategy utilized by many bacteria growing in dynamic environments, such as WWTPs, allowing them to adapt to changing conditions, cycles, or environmental stress (Hernández et al., 2008). The storage compound PHA is common in many bacteria (Sakai et al., 2015). PHA, glycogen, and poly-P are common in polyphosphate-accumulating organisms (PAOs) (Oehmen et al., 2007) although some PAOs, such as *Tetrasphaera*, only contain high levels of poly-P (Fernando et al., 2019). By removing phosphate from activated sludge, this diverse functional group of bacteria performs an important role in WWTPs carrying out enhanced biological phosphate removal. All samples were taken from the aeration tanks, where typical PAOs are expected to have a high level of poly-P and glycogen, and lower, but detectable, PHA (Fernando et al., 2019). Bacterial cells from families *Holophagaceae*, *Thermoanaerobaculaceae*, *Vicinamibacteraceae*, and placeholder families midas_f_502, midas_f_973, midas_f_1548, and midas_f_1096 all had peaks for poly-P and glycogen, but not for PHA (Supplementary Figure 3 and Supplementary Table 3). Some *Blastocatellaceae* cells had peaks only for glycogen, and none of the three storage polymers were detected for the “*Solibacteraceae*” cells (Supplementary Table 3). Raman analyses on the pure cultures of *B. fastidiosa* and *G. fermentans* showed storage of glycogen but not poly-P and PHA.

### Genomes Show Evidence of Aerobic and Anaerobic Respiration, Nitrate Reduction, Iron Reduction, and Polyphosphate Accumulation

**FIGURE 5:**
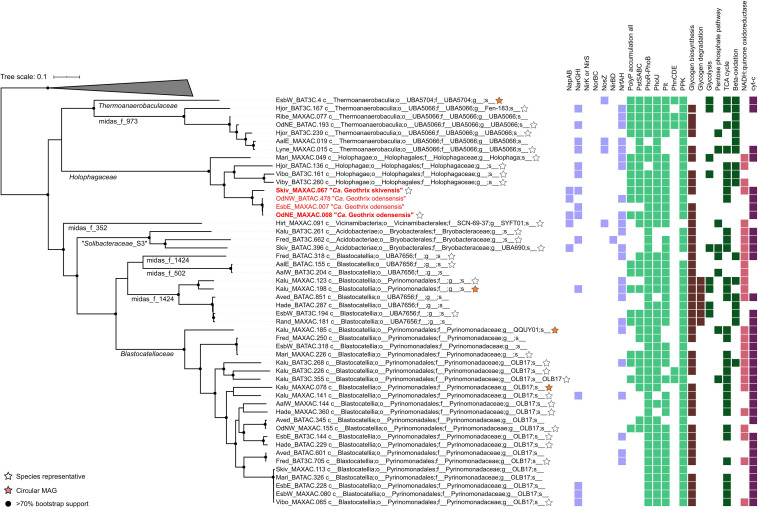
Phylogenetic maximum likelihood genome tree based on the concatenated alignment of 120 single copy proteins created by GTDB-Tk with GTDB RefSeq release 95. Five Chloroflexota genomes were used as the outgroup. Stars indicate the species representatives after 95% ANI clustering. Orange stars indicate the circular MAGs. Bootstrap support >70% is shown by the solid black circles. KEGG modules and genes are indicated by the presence/absence of colored squares (see Supplementary Table 4 for specific KOs). The two novel *Geothrix* species are highlighted in red, with the species representatives bolded. *Solibacteriaceae*_S3 is “*Solibacteriaceae*” subgroup 3 in MiDAS 3 taxonomy.

Analysis of genomic potential based on KO annotations, and the presence of the full set of pathway genes in defined KEGG modules, revealed a diverse range of metabolisms. Here, we have focused on functions of special importance to key wastewater treatment processes. The majority of the “Acidobacteriota” MAG metabolisms represent facultative anaerobic chemoorganoheterotrophs, utilizing various organic compounds including glucose, xylose, acetate, and fatty acids (Supplementary Table 4). The potential for glucose processing through glycolysis (Embden-Meyerhof pathway, KEGG module: M00001) and the pentose phosphate pathway (M00004) was encoded in eight and nine MAGs, respectively, whereas no MAGs encoded the Entner-Doudoroff pathway (M00008). Fermentation to lactate and ethanol was encoded by 35 and nine MAGs, respectively, and the use of fatty acids was identified through genes belonging to beta-oxidation (M00087) in 17 MAGs (Figure 5 and Supplementary Table 4).

Further indications of facultative anaerobic lifestyles were determined based on the presence of a complete TCA cycle (M00009) in 32 MAGs, and cytochrome c oxidase (M00155) identified in 36 MAGs (Figure 5). Alternative electron acceptors under anaerobic conditions include nitrate, with potential dissimilatory nitrate reduction to nitrite (*narGHI*) identified in 15 MAGs (Figure 5). Dissimilatory nitrate reduction to ammonia using *napAB* or *narGHI*, and the respiratory nitrite reductase *nrfAH* was encoded in 11 MAGs, with assimilatory nitrate reduction to ammonia indicated by one MAG encoding *nirBD* (Figure 5 and Supplementary Table 4). Genes for nitrite reduction to nitric oxide (*nirK* or *nirS*) and nitric oxide reduction to nitrous oxide (*norBC*), were not detected in any of the MAGs, revealing that there are no complete denitrifiers in the “Acidobacteriota” MAG set. The absence of *norBC* genes in the genomes suggests that nitric oxide is not used in respiration for these lineages. Overall, the use of oxidized nitrogen species in anaerobic respiration was hypothesized to be uncommon in the “Acidobacteriota” (Eichorst et al., 2018). However, the presence of nitrate reductase genes *narGHI* in 15 MAGs, as well as the *nrfAH* in 23 MAGs indicates that while perhaps uncommon, several lineages likely use nitrate and nitrite in respiration. Potentially, nitrous oxide could also be used as an electron acceptor by four MAGs encoding *nosZ*, indicating the possible reduction of nitrous oxide to nitrogen gas (Figure 5).

Large proteins with multiple CXXCH peptide motifs (≥8) were identified in all the MAGs. The presence of these motifs indicate c-type multiheme cytochromes, which are involved in a wide variety of electron transfer reactions by reducing substrates such as nitrite, sulfite or iron (Edwards et al., 2020). The substrates of the majority of multiheme cytochromes encoded by the MAGs are unknown, and would require targeted experiments on isolates or enrichments to identify. It is important to our understanding of wastewater treatment to determine if “Acidobacteriota” populations are capable of Fe(III) reduction, a so far poorly described process in these systems. There is a substantial pool of oxidized iron in most activated sludge plants, and reduction is known to take place under anaerobic conditions (Rasmussen and Nielsen, 1996), with impact on binding of phosphate in chemical precipitates (Caccavo et al., 1996) and the flocculation properties (Wilén et al., 2000). The potential for iron reduction has been shown in the isolates of *Geobacter sulfurreducens* (Reguera et al., 2005) and *G. fermentans* (Coates et al., 1999). For isolates from *Acidicapsa* it has been shown that they can catalyze the reductive dissolution of ferric iron minerals such as schwertmannite under micro-oxic conditions (Falagán et al., 2017).

Four MAGs appear to belong to genus *Geothrix* based on their ANIs to other *Geothrix* spp. of 82–85% (Supplementary Table 2), and could make excellent candidates for isolation and further tests to determine whether or not they have Fe(III) reduction activity similar to *G. fermentans*. Overall, the diverse metabolisms and the range in genome sizes (3.3–7.7 Mbp) are reflective of the phylogenetic breadth of the “Acidobacteriota” MAGs recovered (Supplementary Table 1).

The accumulation of large amounts of polyphosphate under aerobic or denitrifying conditions is a unique trait of PAOs (Oehmen et al., 2007). The full set of known genes encoding for polyphosphate accumulation (*pitA*, *pstABCS*, *phoU*, and *ppk*), were identified in 21 of the 50 MAGs (Figure 5), supporting the physiology determined through FISH-Raman for cells from the families *Holophagaceae*, *Thermoanaerobaculaceae*, *Vicinamibacteraceae*, and placeholder families midas_f_502, midas_f_973, and midas_f_1548. In line with the FISH-Raman results, all four *Geothrix* MAGs encoded all genes for polyphosphate accumulation, except for one MAG missing the high-affinity phosphate transporter PstABCS, which is likely non-essential to the PAO phenotype (McIlroy et al., 2014) (Figure 5). The low-affinity Pit phosphate transporter is suggested to be essential, though not unique, to the PAO phenotype as the gene is encoded by all known PAOs (Albertsen et al., 2016), but also present in many non-PAO bacteria (Wang et al., 2014). The PHA pathway was incomplete for all of the MAGs, including the *Geothrix* spp., with only *phaA* and *phaC* present in three of the 50 MAGs. This could explain why PHA was not detected in the activated sludge samples by FISH-Raman. However, whether these three lineages have undescribed genes fulfilling the missing functions for PHA accumulation warrants further investigation. Glycogen biosynthesis genes (*glgABC*) were identified in 42 MAGs (KEGG module M00854), and glycogen degradation (*glgP*, *malQ*, *pulA*, and *pgm*) genes were encoded by six MAGs (M00855) (Figure 5). The presence of *glgABC* also supported the results from FISH-Raman where cells from all groups except *Blastocatellaceae*; *Stenotrophobacter*, *Blastocatellaceae*_ASV921, and “*Solibacteraceae*” contained glycogen (Figure 5 and Supplementary Table 3).

**FIGURE 6:**
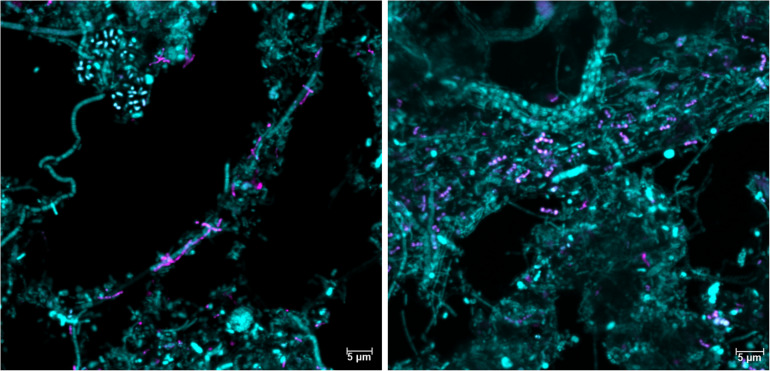
Fluorescence *in situ* hybridization images of the two new *Geothrix* species, “*Candidatus* Geothrix skivensis” **(left)** and “*Candidatus* Geothrix odensensis” **(right)**. They are visualized in activated sludge samples only containing that specific candidatus species in the genus *Geothrix* (as evaluated by amplicon sequencing) using the genus-specific probe Geo_662 (magenta) and the general probe EUB-mix (cyan).

It is well-established that taxonomic classification, naming, and the designation of genomic type material of uncultivated lineages is needed for scientific communication and consistency, as these populations form the majority of the global microbial community (Chuvochina et al., 2019; Konstantinidis et al., 2020). We identified 34 novel “Acidobacteriota” species with MAGs in this study and have formulated names for the two novel *Geothrix* species that fulfill the following criteria: a high-quality MAG, abundant in several WWTPs, functional annotation as determined by the genome and FISH-Raman, and *in situ* morphology as determined by FISH. We propose the names “*Candidatus* Geothrix skivensis” for Skiv_MAXAC.067 (midas_s_201) and “*Candidatus* Geothrix odensensis” for OdNE_MAXAC.008 (midas_s_443) (Figure 5 and Supplementary Figure 4), pertaining to the locations of the WWTPs from which the MAGs were assembled. Specific FISH images show “*Candidatus* Geothrix skivensis” as rod shaped with a length of up to 5 μm and diameter of 0.3–0.5 μm, and “*Candidatus* Geothrix odensensis”, also rod-shaped with a diameter of 0.3–0.5 μm and growing in short chains (Figure 6). See a comprehensive description of the two species in the protologue (Supplementary Table 5).

### The Role of “Acidobacteriota” in the Activated Sludge Ecosystems

“Acidobacteriota” showed a high diversity across the WWTPs investigated with representatives for almost all known lineages in the phylum. In addition, 8 *de novo* families and approx. 23 genera were discovered, adding new diversity to this phylum. This very high diversity likely represents immigration from a variety of natural and engineered habitats collected in the sewer systems (Wu et al., 2019), so their function in the WWTPs is determined by their capability to grow and be active in these systems. The WWTPs are characterized by alternating aerobic and anaerobic conditions and a complex mixture of organic substrates combined with cycling of N and P. We have previously shown that some taxa in “Acidobacteriota” grow in these WWTPs (Saunders et al., 2016), so the high abundance of some genera (e.g., *Geothrix* and Subgroup 10) suggests that they are involved and likely important for the carbon turnover. Besides aerobic respiration, the genomic potential suggests involvement in nitrate reduction (to nitrite or ammonium), which is a key process in the plants.

Annotation of the “*Candidatus* Geothrix skivensis” (Skiv_MAXAC.067, midas_s_201) and “*Candidatus* Geothrix odensensis” (OdNE_MAXAC.008, midas_s_443) MAGs suggest that both may be able to utilize sucrose (K01187), lactate (K00102), and acetate (K00625, K00925). In addition, OdNE_MAXAC.008 was predicted based on the genome to be able to reduce nitrate to ammonia similar to *G. fermentans* (Eichorst et al., 2018). Experimental confirmation is required to determine whether or not these genes are expressed, and potential utilized. FISH-Raman investigations of the abundant taxa and their representative MAGs indicated that some could be involved in the removal of P, either as conventional PAOs with dynamic P-cycling during anaerobic-aerobic conditions, or as non-conventional PAOs accumulating large amounts of poly-P without cycling. Other studies have shown indication of polyphosphate accumulation in “Acidobacteriota” (Foesel et al., 2016), as “Acidobacteriota” were found to contain small granules composed of iron and phosphorus (Wüst et al., 2016). Either way, they seem to be among the increasing list of PAOs to consider for P-removal in WWTPs (Petriglieri et al., 2020), but more studies on their ecophysiology and importance are needed.

The potential fermenting lifestyle of many “Acidobacteriota” combined with tolerance to higher temperatures, such as for *T. aquaticum* (Losey et al., 2013), also would allow some to survive in the anaerobic digesters (commonly present at WWTPs for bioenergy production), when fed with primary or surplus sludge. Surveys have shown high abundances of “Acidobacteriota” in mesophilic anaerobic digesters (Kirkegaard et al., 2017), highlighting the general importance of this phylum in wastewater treatment ecosystems.

## Conclusion

Members of the “Acidobacteriota” are abundant (up to 5%) and widespread in Danish WWTPs with nutrient removal. A high diversity of novel lineages was identified with 8 novel families and approx. 23 genera, and 34 novel “Acidobacteriota” species were discovered based on HQ-MAGs. Ecosystem-specific 16S rRNA gene FISH-probes were designed to target four known and five novel families. These probes revealed different morphologies and mostly small cells (0.5–1.7 μm). The cells were either widely dispersed or found deep within the activated sludge flocs.

Fluorescence *in situ* FISH-Raman revealed intracellular storage of poly-P and glycogen in six of nine families indicating involvement in P-removal, which was supported by annotations of HQ-MAGs. The “Acidobacteriota” MAGs revealed potential for utilizing various organic compounds including glucose, xylose, acetate, and fatty acids. Alternative electron acceptors were also found in many of the MAGs, with potential dissimilatory nitrate reduction to nitrite and ammonia.

We propose names for two novel species, “*Candidatus* Geothrix skivensis” and “*Candidatus* Geothrix odensensis”, based on abundance in WWTPs, recovery of a high-quality MAG, *in situ* morphology and determination of ecophysiological traits. With widespread and high abundance, potential for polyphosphate and glycogen accumulation, nitrate reduction and fermentation, “Acidobacteriota” are important but currently overlooked members of WWTPs warranting further research.

## Etymology

Proposal for the novel species “*Candidatus* Geothrix skivensis” sp. nov. and “*Candidatus* Geothrix odensensis” sp. nov.

“*Candidatus* Geothrix skivensis” (skiv.en’sis. N.L. fem. adj. skivensis pertaining to the city of Skive, the city where the sample origin of the MAG was obtained). This taxon was represented by Skiv_MAXAC.067. Provisional data can be found in Supplementary Table 5.

“*Candidatus* Geothrix odensensis” (o.den.sen’sis. N.L. fem. adj. odensensis pertaining to Odense, a city in Denmark where the sample origin of the MAG was obtained). This taxon was represented by OdNE_MAXAC.008. Provisional data can be found at Supplementary Table 5.

## Data Availability Statement

Amplicon sequencing data are available at the European Nucleotide Archive (https://www.ebi.ac.uk/ena) under study accession number PRJEB42899. The metadata for amplicon sequencing data are available at GitHub (https://github.com/jan niemunk/Publications/tree/master/2021Acidobacteriota). The full length 16S rRNA gene sequences used to perform probe design are available at the European Nucleotide Archive under project number PRJEB26558. Accession numbers for the metagenome assemblies can be found in Supplementary Table 1.

## Author Contributions

JK, CS, and PN designed the study and wrote the manuscript. JK performed 16S rRNA gene phylogenetic analyses and designed the FISH probes. JK and LC performed the FISH probe optimization, imaging, and qFISH. JK and CS performed the metagenomic analyses. FP performed the FISH-Raman analyses. All authors reviewed and approved the final manuscript.

## Conflict of Interest

The authors declare that the research was conducted in the absence of any commercial or financial relationships that could be construed as a potential conflict of interest.

## Footnotes

1 http://www.rstudio.com/

2 https://github.com/MadsAlbertsen/ampvis2

3 https://github.com/geronimp/enrichM

4 github.com/geronimp/HandyScripts/blob/master/99_random/fastacxxch. count.py
