# Supplementary material for: High Diversity and Functional Potential of Undescribed “Acidobacteriota” in Danish Wastewater Treatment Plants
Source: Front Microbiol. 2021 Apr 22;12:643950. doi: 10.3389/fmicb.2021.643950 (PMC8100337; doi:10.3389/fmicb.2021.643950)
Supplement: Supplementary file 2 [file Data_Sheet_1.PDF]

Supplementary Material

High diversity and functional potential of undescribed "Acidobacteriota" in Danish wastewater treatment plants

Jannie Munk Kristensen, Caitlin Singleton, Lee-Ann Clegg, Francesca Petriglieri, Per Halkjær Nielsen

Content:

Figure S1. Phylogenetic 16S rRNA gene tree showing the members of the "Acidobacteriota" found in activated sludge combined with type strain sequences from the Silva v. 138 database.

Figure S2. FISH images of representative cells from "Acidobacteriota" families. The genus or family-specific probes are shown in red and the universal EUB-mix probe is shown in green.

Figure S3. Raman spectrum of *Geothrix* cells.

Figure S4. Heatmap of average abundance of "*Ca.* Geothrix skivensis" (midas_s_201) and "*Ca.* Geothrix odensensis" (midas_s_443).

Tables in excel file (TableS1S2S3S4.xlsx):

Table S1. Classification and statistics of the identified MAGs from Singleton et al. (2020).

Table S2. Genome classifications from GTDB-Tk including ANI to the closest member in the GTDB database.

Table S3. Overview of intracellular polymers found with FISH-Raman.

Table S4. Analysis of the genomic potential based on KEGG Orthology (KO) annotations.

Table S5. Protologues for "*Candidatus* Geothrix skivensis" and "*Candidatus* Geothrix odensensis"

1 Supplementary Figures

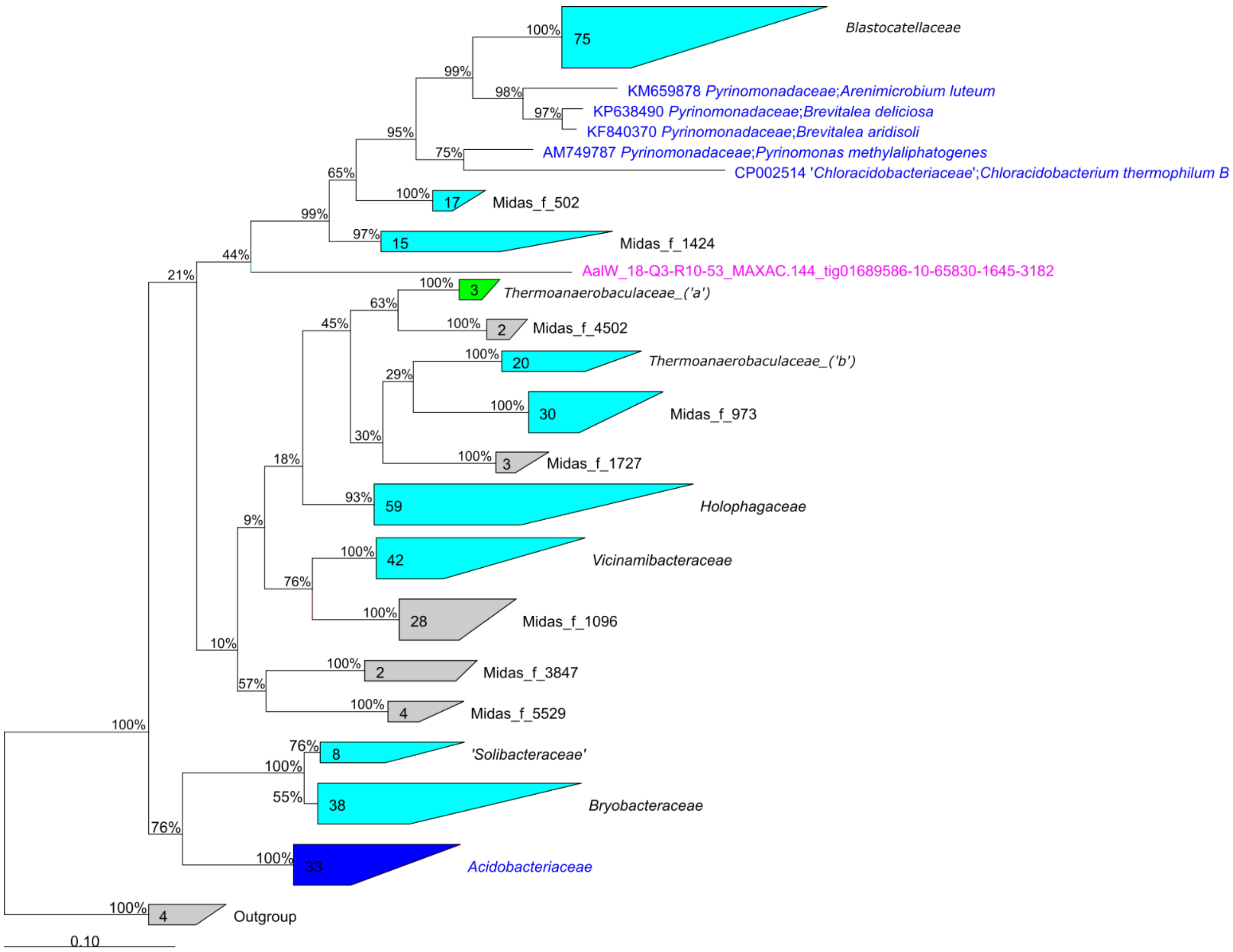

Figure S1 Phylogenetic 16S rRNA gene tree showing the members of the "Acidobacteriota" in Danish activated sludge plants combined with type strain sequences from the SILVA v. 138 database. Labels show the family names of "Acidobacteriota". Unknown *denovo* families are displayed with *denovo* MiDAS identifier (placeholder) names. Groups in grey color contain only MiDAS sequences, blue groups contain only sequences from SILVA v. 138, and green groups contain a combination of MiDAS and SILVA v. 138. Groups colored in cyan represent families that include at least one 16S rRNA gene sequence from MAGs. Only one 16S rRNA gene sequence from MAGs does not fall into clades that include MiDAS sequences (shown in purple). MiDAS sequences from the phylum Nitrospirota are used as an outgroup. Bootstrap values for 1000 replicates are indicated by percentage values.

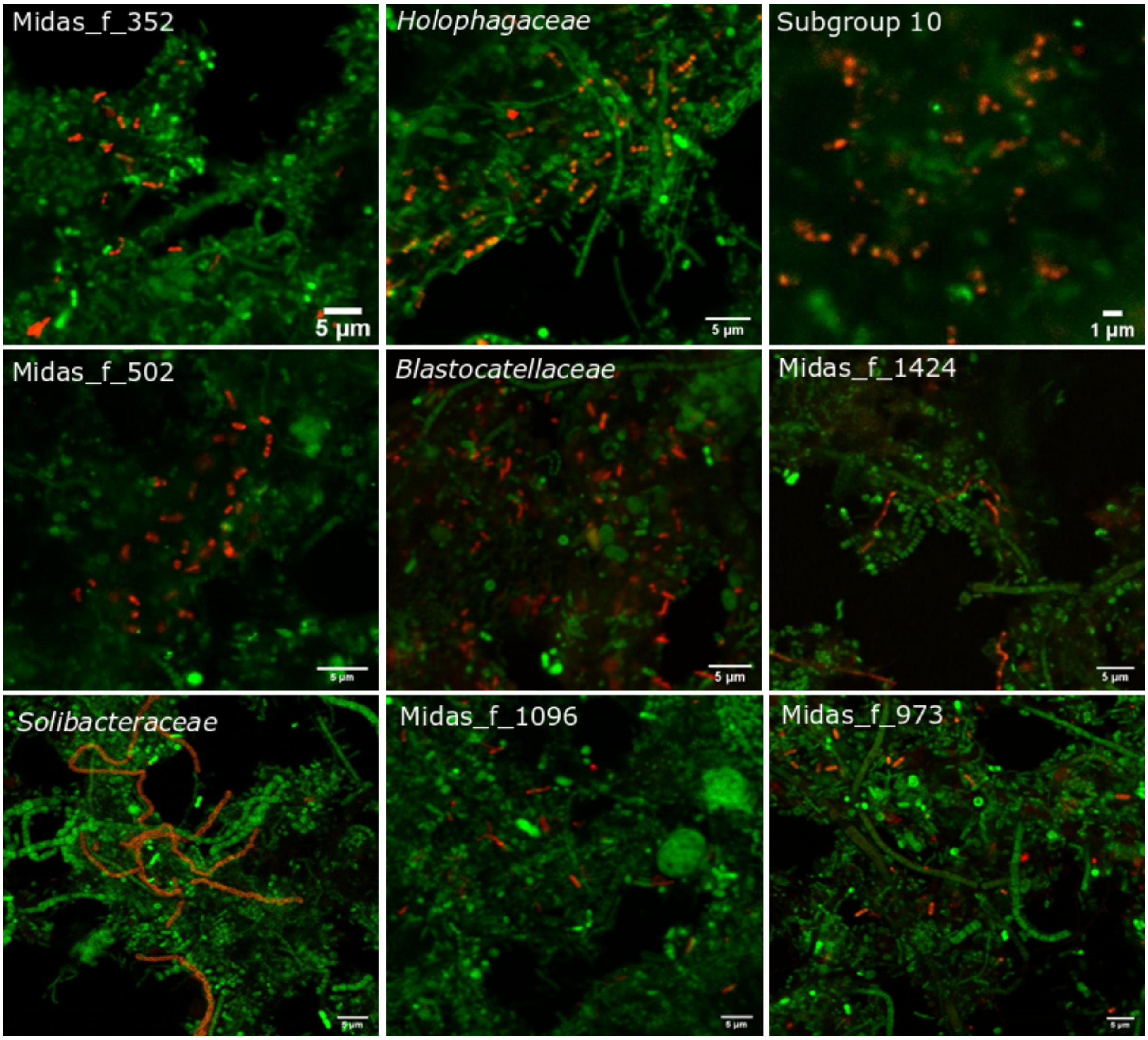

Figure S2. FISH images of representative cells from "Acidobacteriota" families and placeholder family level lineages (Figure S1). The genus or family-specific probes are shown in red, and the universal EUB-mix probe is shown in green. The images were taken using specific probes as listed in Table 1; Midas_f_352 is imaged with the probe Mb2424_1414, *Holophagaceae* by using the probe Holo_1154, *Thermoanaerobaculaceae*; subgroup 10 is imaged with the probe Acido-1162, Midas_f_502 is targeted with the probe DS-100_138, *Blastocatellaceae* is imaged with the probe Blasto_312, Midas_f_1424 is imaged with the probe F11-24_480, "*Solibacteraceae"* is imaged with the probe Soli_499, Midas_f_1096 by using the probe Btb_22_485, Midas_f_973 with the probe f_973_819.

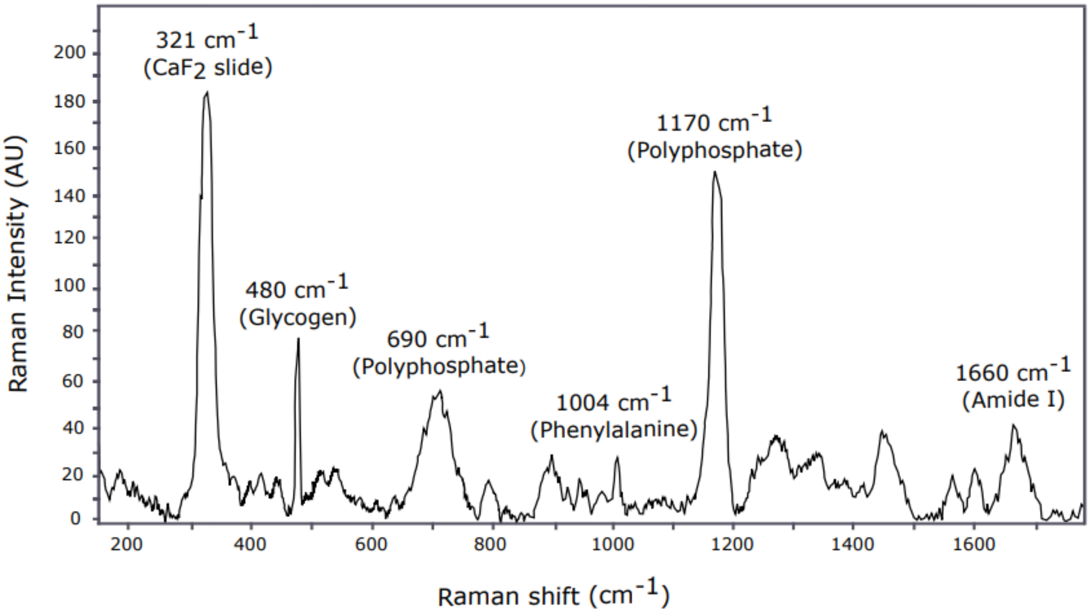

Figure S3. Raman spectrum of *Geothrix* cells from activated sludge (average of 50 FISH-defined cells) showing the presence of signature peaks for glycogen (480 cm^{-1}) and polyphosphate (690 and 1170 cm^{-1}). Peaks for phenylalanine (1004 cm^{-1}) and amide I peptide linkages (1660 cm^{-1}) are used as biological markers.

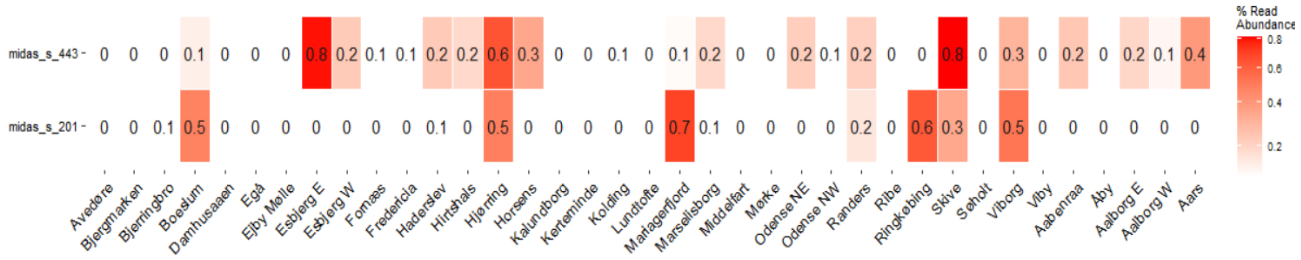

Figure S4. Heatmap of average read abundance of "*Ca.* Geothrix skivensis" (midas_s_201) and "*Ca.* Geothrix odensensis" (midas_s_443) in Danish WWTPs.

**Table S5: Protologues for "*Candidatus* Geothrix skivensis" and "*Candidatus* Geothrix odensensis"**		
**Taxonumber**	N/A	N/A
**Species name**	*Candidatus* Geothrix skivensis	*Candidatus* Geothrix odensensis
**Genus name**	*Geothrix*	*Geothrix*
**Specific epithet**	skivensis	odensensis
**Type species of the genus**	*Geothrix fermentans*	*Geothrix fermentans*
**Taxonumber of the type species**	N/A	N/A
**Genus status**	Validly published	Validly published
**Species etymology**	skiv.en'sis. N.L. fem. adj. skivensis pertaining to the city of Skive, the city where the sample origin of the MAG was obtained	o.den.sen′sis. N.L. fem. adj. odensensis pertaining to Odense, a city in Denmark where the sample origin of the MAG was obtained
**Species status**	sp. nov.	sp. nov.
**Designation of the type MAG**	GCA_016721195.1	GCA_016714685.1
**MAG/SAG accession number**	GCA_016721195.1	GCA_016714685.1
**Genome status**	High-quality draft	High-quality draft
**Genome size**	3749529	3466241
**GC mol %**	67	67.6
**Country of origin**	Denmark	Denmark
**Region of origin**	Skive	Odense
**Source of sample**	Full-scale biological nutrient removal wastewater treatment plant	Full-scale biological nutrient removal wastewater treatment plant
**Geographical location**	Skive	Odense
**Latitude**	56.565132 N	55.432604 N
**Longitude**	9.042158 E	10.458855 E
**Depth**	N/A	N/A
**Altitude**	N/A	N/A
**Temperature of the sample [In celcius degrees]**	Mesophilic	Mesophilic
**pH of the sample**	≈ 7	≈ 7
**Relationship to oxygen**	Facultative anaerobe	Facultative anaerobe
**Energy metabolism**	Potentially utilizing glucose, xylose, acetate and amino acids	Potentially utilizing glucose, xylose, acetate and amino acids
**Assembly**	1 sample	1 sample
**Sequencing technology**	Oxford Nanopore and Illumina HiSeq X	Oxford Nanopore and Illumina HiSeq X
**Binning software used**	Maxbin v2.2.7	Maxbin v2.2.7
**Assembly software used**	CANU v1.8	CANU v1.8
**Habitat**	Full-scale biological nutrient removal wastewater treatment plant	Full-scale biological nutrient removal wastewater treatment plant
**Miscellaneous, extraordinary features relevant for the description**	Elongated rod-shaped morphology	Rod-shaped arranged in chains
